# Simulation and Optimization of FEV Limit Discharge’s Heat Dissipation Based on Orthogonal Experiments

**DOI:** 10.3390/ma13235563

**Published:** 2020-12-06

**Authors:** Hong Li, Yilun Xu, Yong Yang, Chenlong Si

**Affiliations:** School of Mechanical Engineering, Yangzhou University, Yangzhou 225000, China; MZ120190653@yzu.edu.cn (Y.X.); youngyong712@163.com (Y.Y.); sichlon@163.com (C.S.)

**Keywords:** FEV, orthogonal experimental method, battery cooling, simulating optimization

## Abstract

The temperature difference between batteries has effects on the performance of the battery packs of electric vehicles (EVs). Therefore, it is necessary to design a battery cooling management system. In order to reduce the maximum temperature difference of the cooling system of the Formula Electric Vehicle (FEV) automobile, the orthogonal experimental design method was adopted in this paper, and the temperature field of the FEV air-cooled cooling system structure under a short-time high-current discharge condition was simulated for many times. The maximum temperature difference after simulating optimization was about 7 K, and the overall optimization degree was close to 40%. The research results showed that the gap between the single battery and the battery pack was very important to heat dissipation.

## 1. Introduction

In recent years, electric vehicles (EVs) have gradually become the main force in the automobile market due to the introduction of emission regulations of traditional fuel vehicles. The safety of the Li-ion battery under abusive conditions is still a technical barrier for electric vehicles [1,2]. Dendrite growth and overcharging can lead to particularly catastrophic thermal failure due to high rates of heat generation [3]. In actual situations, local high temperature can also cause a short circuit inside the battery, further increasing the temperature and increasing the risk of thermal runaway [4].

In regard to EVs with a lithium-ion battery pack as the main power, the performance of the battery pack mainly depends on the temperature difference between cells [5]. Overheating of the battery may adversely affect the operation, durability, and life of the battery components [6]. Therefore, a battery thermal management system is essential for EVs running on a uniform battery temperature under various conditions [7], which aims to maintain an ideal average temperature between battery cells [8].

In the battery thermal management system, the common ones are air-cooling heat dissipation [9,10], liquid cooling heat dissipation [11,12], phase change material heat dissipation [13,14,15], and heat pipe heat dissipation [16,17,18,19]. However, air cooling is widely used in the Formula Electric Vehicle (FEV) with limited funds due to its simple structure and low cost. Air cooling relies on the continuous flow of cold air to pump the heat generated by the battery pack into the surrounding environment for better cooling performance [20]. Mahamud et al. [7] used a 2D CFD model to conduct numerical simulation analysis on the temperature influence of a cylindrical lithium ion battery under the action of reciprocating air flow. The research results showed that the reciprocating air flow could reduce the temperature of the battery system by 4 K in 72% of the batteries, and the temperature of the highest single battery could be reduced by 1.5 K compared with the one-way air flow. By comparing the hybrid passive thermal management system with the active thermal management system, Kermani et al. [21] found that although the actively forced air convection reached a stable state below the safe temperature at 24 °C, the active and passive systems were ineffective at 40 °C.

In order to promote the development of battery-based new energy vehicles, Formula Student Electric China competition (FSEC) was jointly organized by the Chinese Society of Automotive Engineers in 2013. The competition items include endurance races and obstacle races. With this platform, more students dedicated to new energy research can take advantage of this opportunity to get more exposure to battery power technology. Due to the extremely fierce FSEC competitions, the internal temperature of the power battery of the electric racing car is also more severe than that of the ordinary passenger car. Therefore, the thermal analysis of the battery at the beginning of the design is particularly important. Since all the participants in the FSEC event are college students, it is necessary to deal with the thermal failure of the power battery and ensure that the manufactured cars comply with safety regulations.

This paper combines the simulations of the heat generation of cells and air-cooling heat dissipation of a battery pack with the discharge test of different power ratios in the race conditions, takes the heat management system of the FSEC battery as the research object, and selects the maximum temperature, the minimum temperature, and the maximum temperature difference as the optimization evaluation indexes to optimize and improve the structure of the air-cooling system.

Finally, the FSEC battery air-cooling system designed in this paper can effectively reduce the temperature accumulation of the battery under various working conditions, providing a certain technical reference for the further design of a pure electric racing car.

## 2. Preliminary Design and Thermal Simulation Analysis of FEV Battery System and Cooling System

### 2.1. FEV Battery System Parameter Design and Heat Dissipation Demand

The power battery is the only source of power for the pure electric formula car. The design of the power battery pack not only considers the driving range and power performance of the car but also takes into account the matching of energy and power. The acquisition of various parameters of the power battery pack is the battery. In this study, a lithium iron phosphate polymer lithium ion single battery was selected, and the related physical parameters are shown in Table 1.

#### 2.1.1. Total Battery Capacity Requirement Design

For the endurance test conditions, a fully charged power battery pack must be able to support the car to run a complete endurance test of 22 km. This article is based on the automobile power balance equation:

(1) $$P_{e}=P_{f}+P_{w}+P_{i}+P_{j}$$

where *P_f_* is rolling resistance power; *P_w_* is air resistance power; *P_i_* is slope resistance power; and *P_j_* is Acceleration resistance power.

The specific expression for calculating the maximum output power in the endurance test condition is as follows:

(2) $$P_{e}=\frac{1}{\eta_{T}}\left(\frac{Gfu_{a}}{3600}+\frac{Giu_{a}}{3600}+\frac{C_{D}Au_{a}{}^{3}}{76140}+\frac{\delta mu_{a}}{3600}\frac{du}{dt}\right)$$

where *η_T_* is the transmission efficiency; *G* is the full-load gravity of the vehicle; *f* is the rolling resistance coefficient; *i* is the slope; *C_D_* is the air resistance coefficient; *A* is the windward area; *δ* is the rotation mass conversion factor; *m* is the full vehicle load Mass; *u_a_* is the speed of the car; and *du/dt* is the acceleration of straight-line driving.

Since there is no slope competition in the competition, the slope resistance power *P_i_* is ignored. Substituting the specific parameter values of each part, the calculated output power is 19.505 kW, and the corresponding driving motor with a continuous power of about 20 kW is selected according to the calculated power.

The required energy requirements are calculated based on the durability test driving mileage, and the calculation expression is as follows:

(3) $$W_{S}=P_{e}t=P_{e}\frac{S}{V_{a}}$$

where *W_S_* is the energy consumed when driving *S* mileage; *V_a_* is the average speed in the endurance race, based on competition experience over the years, and the empirical speed design value of it is taken as 60 km/h. Substituting the parameter values of each part, the calculation solution shows that the battery energy required during the durability test is 7.152 kWh. Satisfying this battery capacity requirement can meet the power requirements of all dynamic project tests in the FSEC competition.

#### 2.1.2. Determination of the Number of Single Cells

Considering the rated input voltage of the motor controller and the rules of the 2019 FSEC competition, this paper designs the total voltage of the battery pack to 320 V to meet various requirements in endurance races.

In order to meet the electric vehicle battery pack voltage to meet the design voltage requirements, a certain number of single cells need to be combined in series. The following formula can be used to calculate the number of single cells that need to be connected in series:

(4) $$N_{S}=\frac{V_{t}}{v_{c}}$$

where *N_S_* is the number of battery packs in series; *V_t_* is the rated voltage of the battery pack; and *V_c_* is the nominal voltage of the battery pack. Substituting various parameters, the solution is *N_S_* ≥ 86.486. Considering the space distribution and mass distribution of the vehicle, two battery boxes are used to hang on both sides of the car. Therefore, the total number of single batteries is determined to be an even number; that is, the value is 88.

When the number of single cells in a series is determined, the number of cells in parallel should also be considered to ensure the rationality of the battery pack design, and relevant calculations should be made according to the following formula:

(5) $$n_{P}=\frac{W_{t}}{N_{S}\times v_{c}\times c_{c}}$$

where *n_P_* is the number of batteries in parallel; *W_t_* is the required power of the battery system to complete the event; and *c_c_* is the rated capacity of the single battery. Substituting various parameters, the solution is *n_P_* ≥ 0.998, and the final n*_P_* value is 1, which is combined with the number of single cells in a series, and finally, the entire battery pack is grouped in 88S1P.

#### 2.1.3. Group Design of Power Battery Pack

After relevant calculations, the 88S1P grouping method is adopted, and the 88 single cells are divided into 12 battery modules, of which there are 10 battery modules composed of eight single cells, and each battery module can provide 0.6512 kWh of the energy, the voltage of a single battery module is 29.6 V; the remaining two are each composed of four single cells, each battery module can provide 0.3256 kWh of energy, and the voltage of a single battery module is 14.8 V. Each battery box has a total of 44 single batteries and a total of six battery modules, of which five battery modules are composed of eight single batteries, and one battery module is composed of four single batteries. The battery box can provide a total of 7.1632 kWh of energy with a rated voltage of 325.6 V, which meets the requirements of the event.

#### 2.1.4. Calculation of Heat Dissipation in Cyclic Durable Discharge Conditions

Since the air-cooled heat dissipation system has the advantages of low manufacturing cost, long service life, simple structure, easy maintenance, etc. [21], so air-cooled forced heat dissipation is adopted in this research.

For polymer lithium-ion batteries, the inside can be regarded as an opaque closed system, and the heat radiation generated by high temperature is basically negligible, but considering the physical factors of the gel electrolyte inside, there will be microscopic particles inside. The heat conduction occurs in motion, and the surface will move relative to the air to transfer heat. When considering the heat transfer on the surface, it can be explained by the definition of convective heat transfer. The formula is as follows:

(6) $$Q=h_{con}A_{b}\Delta t$$

where *Q* is the heat flow generated by convective heat transfer, W; *h_con_* is the convective heat transfer coefficient, W/m^2^·K; *A_b_* is the battery heat dissipation surface area, m^2^; and *Δt* is the temperature difference between fluid and solid, K.

The convective heat exchange between the surface of the power battery pack and the air in contact with each other follows Newton’s law of cooling. The internal heat of the cell is transferred to the surface of the aluminum–plastic film and follows the Fourier’s law of heat transfer [22], so the total heat generation can be regarded as the total heat dissipation required by the system, which can be determined by the following formula:

(7) $$Q_{M}=q_{M}V_{M}$$

where *Q_M_* is the total calorific value of the power battery pack, kW; *q_M_* is the heat generation rate of the power battery pack, kW/m^3^; and *V_M_* is the volume of the power battery pack, m^3^.

When a system reaches a stable state, the heat generated by its thermal balance should be equal to the heat dissipated. The air volume required by the forced air cooling system is calculated by the following formula:

(8) $$L_{all}=\frac{Q_{M}}{\rho_{Air}C_{Air}\Delta t}$$

where *L_all_* is the total air flow required for heat dissipation of the power battery cooling system, m^3^/s; *ρ_Air_* is the density of air, taken as 1.293 kg/m^3^; *C_Air_* is the specific heat capacity of air, taken as 1.005 kJ/(kg·K); *Δt* is the temperature difference between the inlet and outlet of the power battery box, K.

The temperature of the air outlet is taken as 38 °C, and the temperature of the air inlet is taken as 22 °C. Substituting the cell body volume 2.006 × 10^−4^ m^2^ and the related heat generation rate data, the heat dissipation air volume required by a single battery is 0.5478 CFM. Comprehensively considering the gap between all the single cells in the power battery box and the number of single cells, it can be seen that the total amount of heat dissipation air required for one power battery box on one side is 24.1014 CFM.

#### 2.1.5. Calculation of Heat Dissipation in Large Current and Short-Time Discharge Conditions

The strong current under the medium and high discharge rate is enough to accumulate a large amount of heat in the battery in a short time, which threatens the battery’s own life and the safety of the entire power battery system. Combined with the heat generation rate and corresponding response of each part of the battery at a discharge rate of 8.9 C, for the effective volume, the simultaneous Equations (7) and (8) can be solved to obtain a heat dissipation air volume of 1.4617 CFM for a single battery, and the total heat dissipation air required for a power battery box is 64.3141 CFM.

#### 2.1.6. Calculation of Air Volume of Cooling Fan

The performance of the cooling fan is directly related to the heat dissipation effect. The air volume is an important factor worth considering when choosing a cooling fan. Generally, the following calculation formula is used:

(9) $$CFM=35.4233\times 60\times S\times V_{Air}$$

where *CFM* is the air volume of the cooling fan, ft^3^/min, which represents the air volume of the fan per minute; *S* is the area of the air outlet, m^2^; and *V_Air_* is the average wind speed of the air, m/s.

Since the cooling system has certain obstacles to the free flow of cooling air, the equipped cooling fan cannot reach its maximum air volume value during actual work. Therefore, in order to alleviate this problem, the maximum air volume value of the cooling fan is selected. This paper chooses two times the calculated air volume value as a consideration [23], and the final total demand air volume is 69.3729 *CFM*. Consider choosing two cooling fans with different air volumes and different sizes to meet the requirements. The cyclone F125 cooling fan has an air volume of 57.32 *CFM* and a specification of 115 mm × 115 mm × 25 mm. The cooling fan model Sanyo DENKI 109P0412J3123 has an air volume of 16.3 CFM and a specification of 35 mm × 35 mm × 28 mm.

### 2.2. Preliminary Thermal Simulation of Power Battery Pack

#### 2.2.1. Establishment of Finite Element Geometric Model

Thermal simulation only considers the battery pack, simplifying the impact of the electrical equipment on the entire system. The connecting copper plates are used to connect single cells in a series, the battery fixing plate is used to fix a single battery pack, the nylon plate is used for insulation, there are various connecting wires, and the rounded corner design of the single cells is ignored.

It is determined that the battery boxes on both sides of the racing car are of a rectangular parallelepiped structure, and the dimensions of the length, width, and height of the master and slave boxes are all 680 mm × 220 mm × 240 mm after preliminary design.

**Figure 1:**
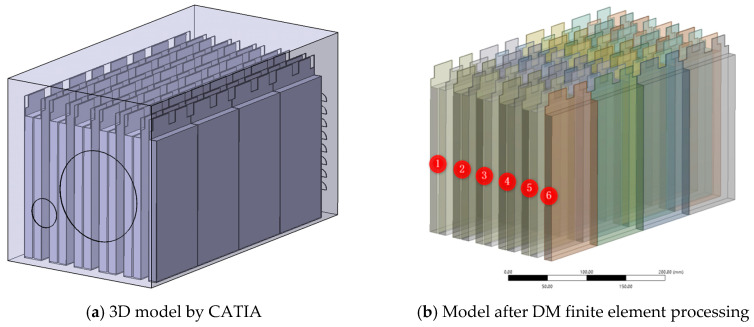
Simplified 3D model of a power battery pack.

**Figure 2:**
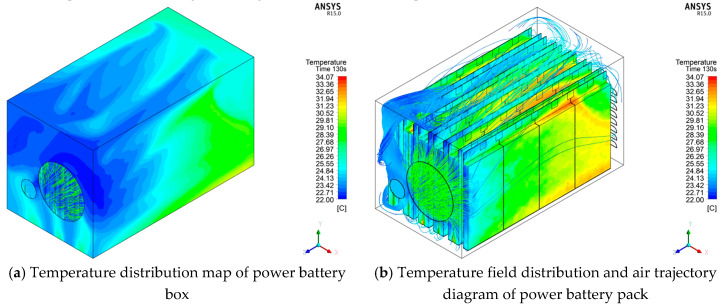
Distribution diagram of cyclic durable discharge condition (130 s).

The simplified three-dimensional model is shown in Figure 1a below, and the position of the air inlet of the cooling fan and the relevant shape and position of the cooling air outlet have been set. The 3D model file is imported into the Design Modeler module and then divided into six parts according to the battery grouping method and corresponding number, as shown in Figure 2b below.

#### 2.2.2. Analysis of Preliminary Thermal Simulation Results of Power Battery Pack

For the preliminarily designed power battery box with an air-cooled device, in order to study the heat dissipation effect of the entire heat dissipation system, the simulation environment was used as the simulation environment for two cycle times of cyclic durable discharge conditions and 45 s of the high-current limit discharge conditions. To simulate the heat generation of the power battery pack, Figure 2 shows the temperature field distribution and the trajectory distribution path results of the cooling air under two cycles of cyclic durable discharge conditions.

Figure 2 shows that when the discharge rate of 5 C is used for two cycles, the maximum temperature of the entire battery pack reaches 34.07 °C, the lowest temperature on the surface of the battery pack is near the cooling fan, the maximum temperature difference in the battery box reaches 12.61 K, and the maximum temperature difference between single cells is 6.53 K. The highest temperature is concentrated in the middle and rear of the power battery pack near the air outlet. Under the action of the cooling fan, the overall temperature of the entire battery pack has dropped, but the discharge is only 130 s. The battery temperature has risen by nearly 12 K compared to the ambient temperature. If the discharge time increases, the overall temperature will inevitably exceed the battery’s normal operating temperature range.

**Figure 3:**
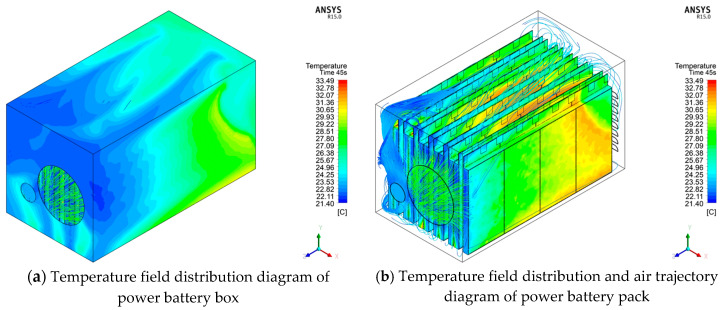
Distribution diagram of short-term high-current limit discharge condition (45 s).

Figure 3 shows the temperature field distribution and the trajectory distribution path results of the cooling air under the short-term high-current discharge condition.

It can be seen from Figure 3 that after 45 s of discharge at a discharge rate of 8.9 °C, the highest temperature of the entire battery pack reaches 33.49 °C, and the lowest temperature still exists on the surface of the battery pack near the cooling fan. The maximum temperature difference reached 12.09 K, and the maximum temperature difference between single cells was 3.38 K. The first half of the entire power battery pack has a low temperature, indicating that the air sent by the cooling fan has performed convective heat exchange well, but the temperature near the air outlet is relatively high. Group physical factors hinder the effect, and the amount of air sent to the middle and rear is very small; second, because the gap between the battery packs makes the air not circulate well, it is not easy to conduct convective heat transfer; third, because the cold air is heated, the hot air moves backward and meets the hot battery. The heat exchange effect between the two cannot reach the expected effect, so for the middle and rear part, especially near the air outlet, the temperature of the battery at the location is relatively high.

## 3. Design of Orthogonal Test Scheme for Heat Dissipation System

The thermal simulation results of the preliminary design of the air cooling heat dissipation system show that the maximum temperature difference of the system is still too large. Considering the heat dissipation defects of the preliminary design structure, it is necessary to seek the key factors and relevant levels that affect the heat dissipation effect of the FEV air cooling heat dissipation system and find the optimal and most reasonable design combination scheme.

In the orthogonal experimental scheme of the FEV air cooling and cooling system, 13 factors in Table 2 are selected as considerations, the parameters of the selection factors in the preliminary design are comprehensively considered, and three levels are reasonably given. After consulting the orthogonal table specifications, an L27 (313) type orthonormal table was selected, and the related factors were numbered alphabetically, which are described in the following text. The maximum temperature, minimum temperature, and maximum temperature difference in the system were selected as the heat dissipation evaluation index.

In Table 2, the single battery pack clearance parameter is given according to the sum of the thickness of the partition board and the clearance reserved in the actual design after simplifying the partition board. The specific horizontal parameter values of each factor are given according to the above table, and the specific design scheme of this FEV orthogonal experiment is given according to the L27 (313) orthogonal table selected. Detailed data are shown in Table 3.

## 4. Evaluation Index Analysis of Heat Dissipation Simulation Results

### 4.1. Analysis of FEV Orthogonal Test Scheme and Factor Results

**Figure 4:**
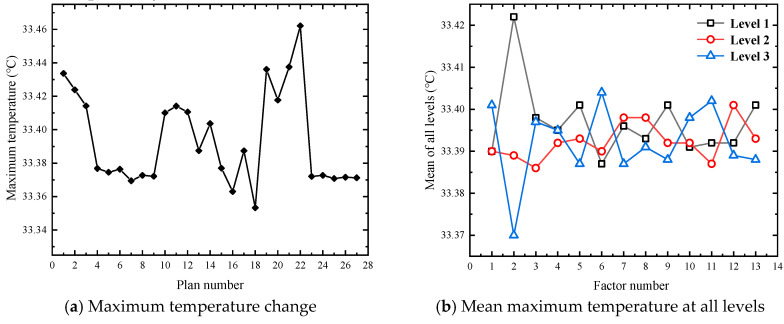
Variation of simulation results of maximum temperature in an orthogonal experiment.

**Figure 5:**
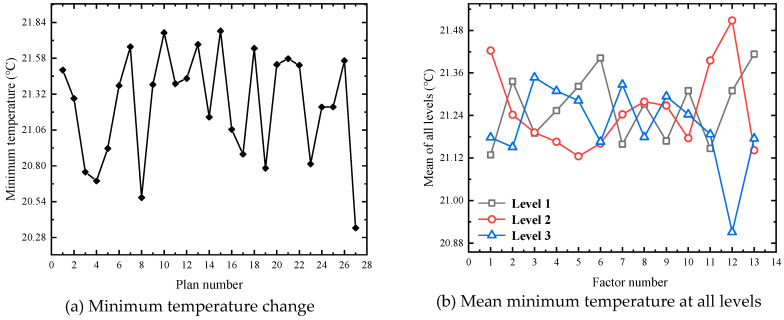
Variation of simulation results of minimum temperature in orthogonal experiment.

**Figure 6:**
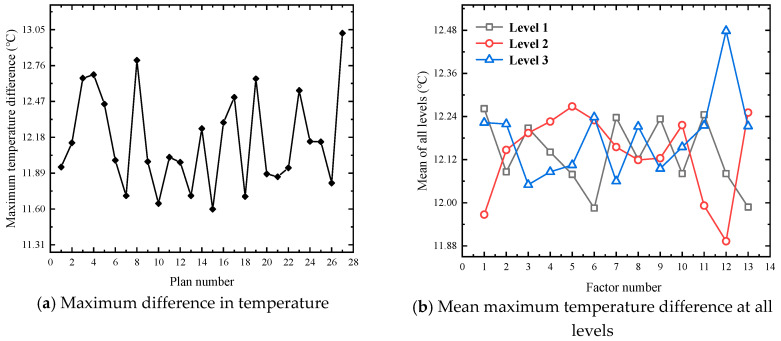
Variation of simulation results of maximum temperature difference in the orthogonal experiment.

Based on the 75 M linear acceleration and high-speed obstacle avoidance tests in FEV competition, the large current under the limit discharge condition is selected for heat dissipation simulation, which can more visually and reliably explore the temperature condition under the limit discharge condition of the battery, and it can also better propose substantive solutions for improving the heat dissipation effect. Therefore, the orthogonal experimental design scheme is based on an 8.9 C limit discharge ratio and short-time large-current discharge 45 s to simulate the temperature conditions under 27 experimental schemes. Figure 4, Figure 5 and Figure 6 show the changes of the maximum temperature, the minimum temperature, and the maximum temperature difference under 27 schemes, respectively.

It can be seen from Figure 4a that there is no significant difference in the value of the maximum temperature, as there is only a difference of 0.109 K. Moreover, it can be seen from Figure 4b that the influence of level 1 and level 3 set under factor 2 on the maximum temperature is significant, with a difference of 0.052 K, while the average maximum temperature fluctuates between 33.385 and 33.405 °C under other factors. According to the range analysis of the average maximum temperature at three levels, the order of the influencing factors is B > F > K > E > I ≈ M > C ≈ L > A ≈ G > H ≈ J > D.

As can be seen from Figure 5a, compared with the maximum temperature, the lowest temperature has a significant numerical change, and the maximum difference between different schemes reaches 1.428 K. It can be seen from Figure 5b that factor 12 has a greater influence on the minimum temperature. Under this factor, the maximum difference between level 2 and level 3 is 0.597 K. According to the range analysis of the mean minimum temperature at three levels, the primary and secondary order of the influencing factors is L > A > M > K > F > E > B > G > C > D > J > I > H.

It can be seen from Figure 6a, similar to the minimum temperature, the maximum temperature difference also shows a relatively large numerical change, with the maximum difference between different schemes reaching 1.423 K. It can be seen from Figure 6b that the factor with a significant impact on the maximum temperature difference and the factor with a significant impact on the minimum temperature is factor 12, and the maximum difference between level 2 and level 3 is 0.585 K. According to the range analysis between the mean maximum temperature difference at the three levels, the primary and secondary influencing factors is L > A > M > F ≈ K > E > G > C > D > I > J ≈ B > H.

Comprehensive analysis of Figure 4, Figure 5 and Figure 6 shows that 27 groups after solution heat simulation of the power battery box of maximum temperature, minimum temperature, and maximum temperature fluctuations from the figure on the reaction are relatively large, and each level of the highest temperature, lowest temperature, and the average of the maximum temperature difference, in addition to the individual factors, vary widely between different levels, while the remaining factors fluctuate within a certain range. The above analysis only gives a rough plan guidance for the average results under various factors, and it only clarifies the primary and secondary relationship of the influencing factors without determining the specific plan parameters. Moreover, the analysis results are based on the average level, which cannot guarantee the consistency in the participating schemes. Therefore, the following part will carry out specific exploration and analysis of the results of a single index for each evaluation index.

### 4.2. Analysis of Thermal Simulation Temperature Effects

In order to analyze the specific relationship between the three horizontal batches corresponding to 13 factors and the simulation temperature results, the trend analysis of the simulation temperature results under each factor level is carried out in order to find the influence trend of the variation of each factor level on the heat dissipation effect of the air-cooling heat dissipation system.

**Figure 7:**
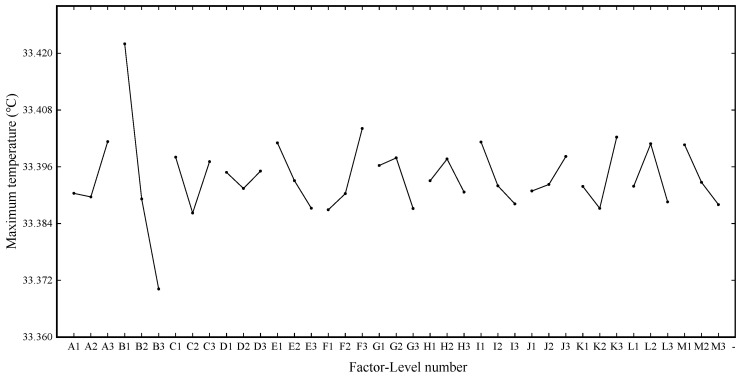
Maximum temperature effect curve.

Range analysis was conducted on the maximum temperature data after horizontal batch simulation calculation under different factors of 27 groups of schemes, and the primary and secondary order of factors affecting this evaluation index was finally determined as B > F > K > E > I > M > L > C > A > G > J > H > D. Figure 7 shows the graphs of the maximum temperature effect.

It can be seen from Figure 7 that factor B (gap of single battery pack) has the most significant influence compared with other factors. For the highest temperature, the smaller the value, the better the heat dissipation effect and the more reasonable the system structure design. The optimal combination under this evaluation index is B3 F1 K2 E3 I3 M3 L3 C2 A2 G3 J1 H3 D2.

**Figure 8:**
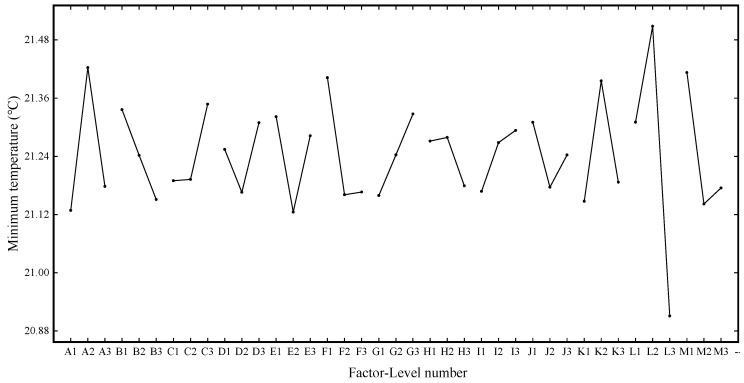
Minimum temperature effect curve.

For the evaluation index of the heat dissipation effect, the lower the value, the better the heat dissipation effect. Range analysis was conducted on the minimum temperature data after horizontal batch simulation calculation under different factors of 27 groups of schemes, and the primary and secondary order of factors affecting this evaluation index were finally determined as L > A > M > K > F > E > B > G > C > D > J > I > H. Figure 8 shows the graphs of the minimum temperature effect.

From the analysis of Figure 8, it can be seen that factor L (X-direction spacing of the second air intake) has the most significant influence on the evaluation index. According to the level of factors reaching the standard and the order of primary and secondary factors affecting the minimum temperature, the optimal combination under this evaluation index can be determined as L3 A1 M2 K1 F2 E2 B3 G1 C1 D2 J2 I1 H3.

**Figure 9:**
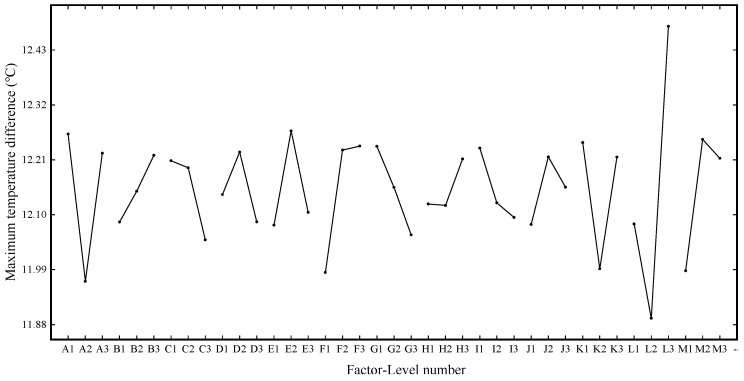
Maximum temperature difference effect curve.

As for the evaluation index of the heat dissipation effect, domestic and foreign scholars hope to reduce the internal temperature in homogeneity of the battery pack to ensure that the power battery works under the optimal ambient temperature and improves the overall performance of pure electric vehicles. This paper also hopes that the value can be as small as possible in order to ensure the temperature uniformity among the individual batteries in the power battery pack. The range analysis was carried out on the maximum temperature difference data after horizontal batch simulation calculation under different factors of 27 groups of schemes, and the primary and secondary order of factors affecting this evaluation index were finally determined as L > A > M > F > K > E > G > C > D > I > J > B > H. Figure 9 shows the graphs of the maximum temperature difference effect.

It can be seen from Figure 9 that the influence of factor L (X-direction spacing of second air intake) on the evaluation index is the same as the minimum temperature. By comparing the minimum values at each factor level in the figure and according to the level of factors reaching the standard and the order of primary and secondary factors affecting the maximum temperature difference, the optimal combination under this evaluation index can be determined as L2 A2 M1 F1 K2 E1 G3 C3 D3 I3 J1 B1 H2.

After analyzing the above three major single evaluation indexes, the influence of various factors and related level parameters on the heat dissipation effect of the FEV power cell air-cooled heat dissipation system is clarified. In addition, in the orthogonal experimental analysis method, there is an F-test, which is commonly known as a joint hypothesis test—also known as variance ratio test or variance homogeneity test. It is a test based on a null hypothesis that statistical values obey F-distribution. Combined with the F ratio in the orthogonal experimental design scheme in this paper, the overall influence degree of each factor on the heat dissipation effect is determined first, and the primary and secondary order is L > B > A > M > F > K > E > C > G > I > D > J > H. Secondly, according to the range difference of each factor at the corresponding level under each evaluation index, the combination obtained is L2 B1 A3 M1 F3 K3 E1 C1 G2 I1 D3 J3 H2.

According to the L27(313) orthonormal table scheme arranged in this paper, the above four optimization schemes are not found. Therefore, it is necessary to conduct a thermal simulation calculation for the above four schemes again, and relevant temperature data results are summarized in Table 4.

By analyzing the data in Table 4, it can be seen that when the combined scheme is L3 A1 M2 K1 F2 E2 B3 G1 C1 D2 J2 I1 H3, the maximum temperature drops significantly, and the maximum temperature difference of 9.5357 K also reaches the minimum value in the proposed scheme. The completion of this evaluation index can effectively maintain the homogeneity of the battery. By comparing the heat dissipation simulation data after the preliminary design, it can be found that the optimized maximum temperature is 1.4376 K lower than the original, and the optimized degree of the maximum temperature difference reaches 21.1270%. For the designed L27(313) orthogonal experimental scheme, after simulation, analysis, and research, the optimized scheme meets the research requirements of the first orthogonal experimental design method in this paper.

### 4.3. Analysis of Multiple Orthogonal Experiment Simulation Optimization Results

In order to further improve the degree of optimization of the FEV battery cooling system, with the aid of the orthogonal experiment design software, combined with the orthogonal test design scheme for the first time under the results, under the consideration and analysis of the cooling effect of the three evaluation indexes of F ratio difference, we picked the most significant factors affecting the second orthogonal simulation test analysis, and so on, repeating to arrange a multiple orthogonal experiment design. The new influencing factors were selected, and the horizontal parameters were changed based on the last optimal scheme parameters. In this way, the structure of the FEV power cell air-cooled heat dissipation system was continuously optimized and improved. Finally, after arranging different orthogonal experimental schemes for three times, an optimal combination scheme was found. The specific parameters of the scheme changed, and the specific program is shown in Table 5.

**Figure 10:**
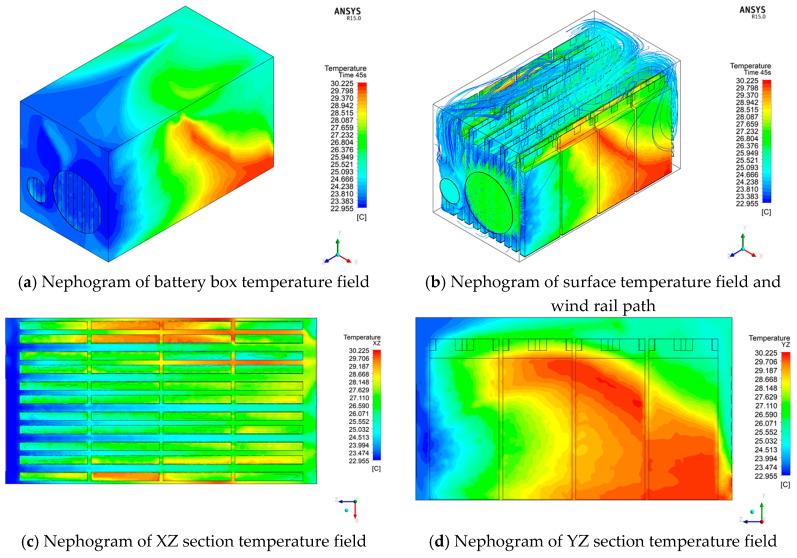
Distribution of discharge temperature in short time (8.9 °C discharge 45 s) with large current.

The temperature field distribution results under this scheme are shown in Figure 10.

As can be seen from Figure 10a,b, when the clearance of a single battery pack under the optimization scheme becomes larger, the clearance between two ends of the power battery box with a fixed width and the power battery pack is too small, so the overall temperature at both ends is slightly higher, but the maximum temperature of the whole system is significantly reduced. It can be seen from the figure that the maximum temperature is 30.2254 °C, and the maximum temperature difference is 7.2704 K. Compared with the maximum temperature difference under the design of the first orthogonal experiment, 23.7559% is optimized. By observing the cloud charts in Figure 10c,d, it can be found that the temperature of battery 1 near the first air inlet is slightly lower than that of the second air inlet. The temperature of the top part of battery 2, the upper part of battery 3, and the lower part of battery 4 far away from the air inlet is high, and the overall heat dissipation effect of battery 3 is not good, which is closely related to the location of the cooling fan and the air volume. Cooling fan 1 is positioned more to the right of the box. Due to the gap between the box on the right and the battery pack being very small, there is poor air flow. Meanwhile, it can be seen from Figure 10b that the air volume on the right is very small, so the heat dissipation effect of the battery on the right is relatively poor. Due to the small air volume of cooling fan 2, the left battery pack cannot get strong cold air to cool the battery, so the heat dissipation effect of the left battery is slightly worse than that of the right battery. However, the overall battery is located in the middle, so the temperature of the overall battery is obviously controlled except for the battery with a poor heat dissipation effect at both ends.

Table 6 shows the comparison results before and after the optimization design of the FEV power battery box air-cooled heat dissipation system.

By analyzing the data in Table 5, it can be found that through a reasonable selection of factors and relevant levels that have significant effects on the heat dissipation system, and through three orthogonal simulation tests, the optimization degree percentage of the maximum temperature difference reaches nearly 40%. In the heat dissipation system, the temperature uniformity is an important index to evaluate the thermal management of the battery. This optimization degree has achieved the purpose of improving the heat dissipation performance of the power battery pack.

**Figure 11:**
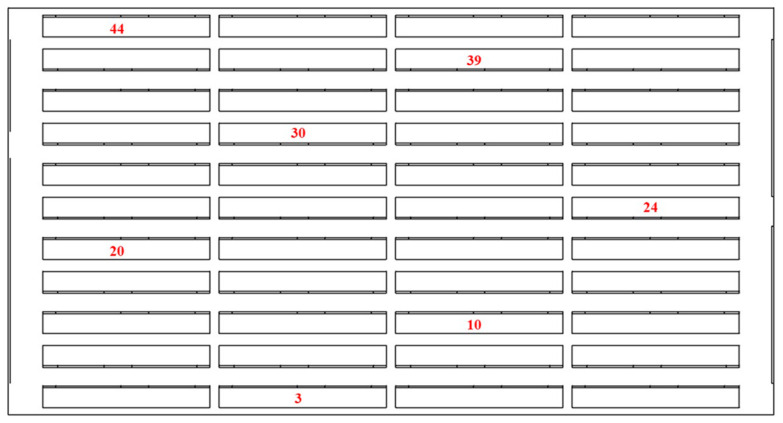
Temperature monitoring layout of the battery pack.

According to the above thermal simulation results of the battery pack under different working conditions, the heat dissipation effect of the optimized air-cooled heat dissipation system was analyzed by mainly monitoring the batteries of some key modules in the battery pack in the test. Figure 11 shows the layout of temperature sensor monitoring in the battery pack, and Figure 11 shows the comparison curve of the test and simulation temperature under the conditions of 8.9 K discharge for 45 s; the first 10 s is the standing temperature.

**Figure 12:**
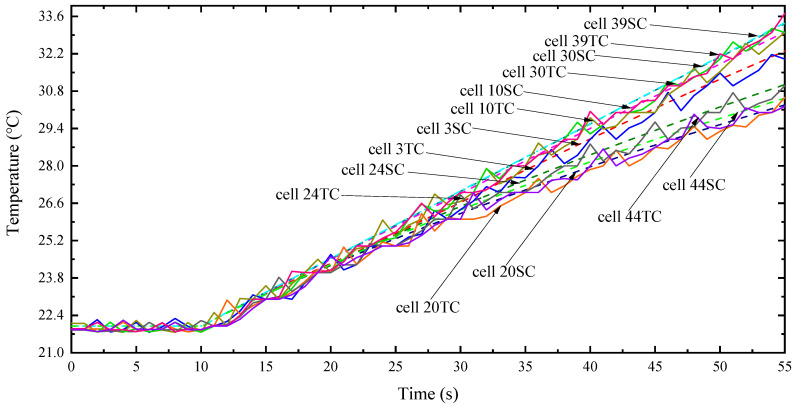
Comparison curve between experiment and simulation.

The analysis in Figure 12 shows that the simulation temperature of the simulation model and the temperature data measured under the temperature test bench are basically relatively close. The maximum error temperature can be stabilized within 3.2 K during the debugging process. The maximum temperature does not exceed the upper limit of 75 °C stated in the battery specification, indicating that the optimized FEV power battery air-cooled heat dissipation system model still has a relatively high credibility, and it also shows that the above optimization can be used. The optimized plan predicts and analyzes the heat dissipation of the FEV power battery pack.

## 5. Conclusions

In this paper, the orthogonal experimental method was used to carry out multiple simulation and optimization analysis of the heat dissipation system based on the preliminary design parameters of the structure of FEV air cooling heat dissipation system, and finally, the optimal scheme was used to improve the structure of the heat dissipation system. The simulation study of FEV events was carried out under the condition of short-term large current discharge, the optimized maximum temperature difference was stable at about 7 K, and the overall optimization degree was close to 40%. The fitting rate of the battery pack temperature monitoring test curve and simulation curve is high, and the influence of the single cell gap and battery pack gap on heat dissipation is very important.

## Figures and Tables

**Table 1:** Physical parameters of a lithium iron phosphate polymer lithium ion single battery.

Item	Rated Parameters
Typical capacity/mAh	22,000
Cell resistance/mΩ	3
Cell thickness/mm	11.8
Cell length/mm	194
Cell weight/g	420
Cell width/mm	91
Nominal voltage/V	3.7
Charge cutoff voltage/V	4.2
Cathode width/mm	35
Anode width/mm	25
Maximum discharge current/A	220
Standard charging current/A	22
Standard discharge current/A	22
Discharge temperature/°C	−20~75

**Table 2:** Factors of the Formula Electric Vehicle (FEV) air cooling system orthogonal test scheme level.

Factors	Alpha Code	Level 1	Level 2	Level 3
Single cell clearance/mm	A	1	2	3
Single battery pack clearance/mm	B	2.5	3.5	4.5
Battery pack clearance/mm	C	10	11	12
Number of outlet grilles/unit	D	16	18	20
Air outlet grille height/mm	E	10	10.5	11
X-direction spacing of outlet grille/mm	F	17	18	19
Y-direction spacing of outlet grille/mm	G	6.5	7	7.5
Air inlet size R1/mm	H	59	60	61
X-direction spacing of first air intake/mm	I	28.5	26.5	24.5
Y-direction spacing of first air intake/mm	J	12.5	14.5	16.5
Air inlet size R2/mm	K	19	20	21
X-direction spacing of second air intake/mm	L	54.5	56.5	58.5
Y-direction spacing of second air intake/mm	M	48	46	44

**Table 3:** Orthogonal experimental design scheme of FEV air-cooling heat-dissipation system.

Number	A	B	C	D	E	F	G	H	I	J	K	L	M
1	1	2.5	10	16	10.0	17	6.5	59	28.5	12.5	19	54.5	48
2	1	2.5	10	16	10.5	18	7.0	60	26.5	14.5	20	56.5	46
3	1	2.5	10	16	11.0	19	7.5	61	24.5	16.5	21	58.5	44
4	1	3.5	11	18	10.0	17	6.5	60	26.5	14.5	21	58.5	44
5	1	3.5	11	18	10.5	18	7.0	61	24.5	16.5	19	54.5	48
6	1	3.5	11	18	11.0	19	7.5	59	28.5	12.5	20	56.5	46
7	1	4.5	12	20	10.0	17	6.5	61	24.5	16.5	20	56.5	46
8	1	4.5	12	20	10.5	18	7.0	59	28.5	12.5	21	58.5	44
9	1	4.5	12	20	11.0	19	7.5	60	26.5	14.5	19	54.5	48
10	2	2.5	11	20	10.0	18	7.5	59	26.5	16.5	19	56.5	44
11	2	2.5	11	20	10.5	19	6.5	60	24.5	12.5	20	58.5	48
12	2	2.5	11	20	11.0	17	7.0	61	28.5	14.5	21	54.5	46
13	2	3.5	12	16	10.0	18	7.5	60	24.5	12.5	21	54.5	46
14	2	3.5	12	16	10.5	19	6.5	61	28.5	14.5	19	56.5	44
15	2	3.5	12	16	11.0	17	7.0	59	26.5	16.5	20	58.5	48
16	2	4.5	10	18	10.0	18	7.5	61	28.5	14.5	20	58.5	48
17	2	4.5	10	18	10.5	19	6.5	59	26.5	16.5	21	54.5	46
18	2	4.5	10	18	11.0	17	7.0	60	24.5	12.5	19	56.5	44
19	3	2.5	12	18	10.0	19	7.0	59	24.5	14.5	19	58.5	46
20	3	2.5	12	18	10.5	17	7.5	60	28.5	16.5	20	54.5	44
21	3	2.5	12	18	11.0	18	6.5	61	26.5	12.5	21	56.5	48
22	3	3.5	10	20	10.0	19	7.0	60	28.5	16.5	21	56.5	48
23	3	3.5	10	20	10.5	17	7.5	61	26.5	12.5	19	58.5	46
24	3	3.5	10	20	11.0	18	6.5	59	24.5	14.5	20	54.5	44
25	3	4.5	11	16	10.0	19	7.0	61	26.5	12.5	20	54.5	44
26	3	4.5	11	16	10.5	17	7.5	59	24.5	14.5	21	56.5	48
27	3	4.5	11	16	11.0	18	6.5	60	28.5	16.5	19	58.5	46

**Table 4:** Thermal simulation results of optimization scheme.

Project Portfolio	Maximum Temperature/°C	Minimum Temperature/°C	Maximum Temperature Difference/K
B3F1K2E3I3M3L3C2A2G3J1H3D2	32.9656	21.9412	11.0244
L3A1M2K1F2E2B3G1C1D2J2I1H3	32.0529	22.5172	9.5357
L2A2M1F1K2E1G3C3D3I3J1B1H2	33.1124	21.2543	11.8581
L2B1A3M1F3K3E1C1G2I1D3J3H2	32.4201	21.2689	11.1512

**Table 5:** Optimal scheme parameters.

Factors	Parameter
Single cell clearance/mm	5
Single battery pack clearance/mm	6.5
Battery pack clearance/mm	10
Number of outlet grilles/unit	18
Air outlet grille height/mm	11
X-direction spacing of outlet grille/mm	17
Y-direction spacing of outlet grille/mm	7
Air inlet size R1/mm	61
X-direction spacing of first air intake/mm	32
Y-direction spacing of first air intake/mm	28
Air inlet size R2/mm	25
X-direction spacing of second air intake/mm	68
Y-direction spacing of second air intake/mm	52

**Table 6:** Comparison of results before and after optimization of the air-cooling system design.

Project	Maximum Temperature/°C	Minimum Temperature/°C	Maximum Temperature Difference/K
Preliminary design	33.4906	21.4007	12.0899
Many optimization	30.2254	22.9550	7.2704
Percentage degree of optimization %	9.7496	7.2628	39.8639
